# Social Determinants of Health for People With Serious Mental Illness After Transitioning to the Community

**DOI:** 10.1093/geroni/igaa057.105

**Published:** 2020-12-16

**Authors:** Kathy Kellett, Kaleigh Ligus, Kristin Baker, Julie Robison

**Affiliations:** 1 UConn Health, Farmington, Connecticut, United States; 2 University of Connecticut, Farmington, Connecticut, United States

## Abstract

Approximately 10 million, or 6 percent, of the U.S. population experience serious mental illness (SMI) (NAMI, 2019). Social determinants of health (SDOH) associated with this population can provide important information for targeted innovations with the potential to reduce disease burden and improve quality of life. Using secondary data from Connecticut’s Money Follows the Person Rebalancing Demonstration, this research compares people age 50+ who transitioned out of an institution onto the Medicaid HCBS Mental Health Waiver (MHW) (n= 271) to those receiving Mental Health services through the Medicaid State Plan (MHSP) (n=278). Analyses examine SDOH in both groups and are organized around five broad domains: Finances; education; social/community context, health/health care, and neighborhood/built environment. MHSP participants were significantly more likely to report not having enough money at the end of the month at 6 (42% vs. 21%), 12 (37% vs. 20%), and 24 (37% vs. 17%) months. Significantly more MHSP than MHW participants did not like where they lived at 6 (12% vs. 1%) and 24 (24% vs. 5%) months. Significantly more MHSP than MHW participants were unhappy with the help they received in the community at 6 (22% vs. 8%), 12 (23% vs. 7%), and 24 (19% vs. 5%) months. Groups did not differ by education, social/community context, health/health care, feelings of safety where they live, or on post-transition hospitalizations, ED use or reinstitutionalization. To improve quality of life in the community, MHSP participants could benefit from greater assistance with finances, housing, and community services.

